# Prediction of extubation outcome in mechanically ventilated patients: Development and validation of the Extubation Predictive Score (ExPreS)

**DOI:** 10.1371/journal.pone.0248868

**Published:** 2021-03-18

**Authors:** Antuani Rafael Baptistella, Laura Maito Mantelli, Leandra Matte, Maria Eduarda da Rosa Ulanoski Carvalho, João Antonio Fortunatti, Iury Zordan Costa, Felipe Gabriel Haro, Vanda Laís de Oliveira Turkot, Shaline Ferla Baptistella, Diego de Carvalho, João Rogério Nunes Filho

**Affiliations:** 1 Universidade do Oeste de Santa Catarina (UNOESC), Joaçaba, Santa Catarina, Brazil; 2 Programa de Pós-Graduação em Biociências e Saúde, Universidade do Oeste de Santa Catarina, Joaçaba, Santa Catarina, Brazil; 3 Hospital Universitário Santa Terezinha, Joaçaba, Santa Catarina, Brazil; Ospedale Sant’Antonio, ITALY

## Abstract

Despite the best efforts of intensive care units (ICUs) professionals, the extubation failure rates in mechanically ventilated patients remain in the range of 5%–30%. Extubation failure is associated with increased risk of death and longer ICU stay. This study aimed to identify respiratory and non-respiratory parameters predictive of extubation outcome, and to use these predictors to develop and validate an “Extubation Predictive Score (ExPreS)” that could be used to predict likelihood of extubation success in patients receiving invasive mechanical ventilation (IMV). Derivation cohort was composed by patients aged ≥18 years admitted to the ICU and receiving IMV through an endotracheal tube for >24 hours. The weaning process followed the established ICU protocol. Clinical signs and ventilator parameters of patients were recorded during IMV, in the end phase of weaning in pressure support ventilation (PSV) mode, with inspiratory pressure of 7 cm H_2_O over the PEEP (positive end expiratory pressure). Patients who tolerated this ventilation were submitted to spontaneous breathing trial (SBT) with T-tube for 30 minutes. Those who passed the SBT and a subsequent cuff-leak test were extubated. The primary outcome of this study was extubation success at 48 hours. Parameters that showed statistically significant association with extubation outcome were further investigated using the receiver operating characteristics (ROC) analysis to assess their predictive value. The area under the curve (AUC) values were used to select parameters for inclusion in the ExPreS. Univariable logistic regression analysis and ROC analysis were performed to evaluate the performance of ExPreS. Patients’ inclusion and statistical analyses for the prospective validation cohort followed the same criteria used for the derivation cohort and the decision to extubate was based on the ExPreS result. In the derivation cohort, a total of 110 patients were extubated: extubation succeeded in 101 (91.8%) patients and failed in 9 (8.2%) patients. Rapid shallow-breathing index (RSBI) in SBT, dynamic lung compliance, duration of IMV, muscle strength, estimated GCS, hematocrit, and serum creatinine were significantly associated with extubation outcome. These parameters, along with another parameter—presence of neurologic comorbidity—were used to create the ExPreS. The AUC value for the ExPreS was 0.875, which was higher than the AUCs of the individual parameters. The total ExPreS can range from 0 to 100. ExPreS ≥59 points indicated high probability of success (OR = 23.07), while ExPreS ≤44 points indicated low probability of success (OR = 0.82). In the prospective validation cohort, 83 patients were extubated: extubation succeeded in 81 (97.6%) patients and failed in 2 (2.4%) patients. The AUC value for the ExPreS in this cohort was 0.971. The multiparameter score that we propose, ExPreS, shows good accuracy to predict extubation outcome in patients receiving IMV in the ICU. In the prospective validation, the use of ExPreS decreased the extubation failure rate from 8.2% to 2.4%, even in a cohort of more severe patients.

## Introduction

Weaning and extubation are critical processes in the management of patients on invasive mechanical ventilation (IMV). The intensive care professional needs to find the ideal balance between unnecessary delay in the discontinuation of IMV—which increases the risk of ventilator-associated complications and hospitalization costs—and premature withdrawal—which could result in extubation failure, difficulty in reestablishing artificial airways, and compromised gas exchange [1].

In the intensive care unit (ICU), spontaneous breathing trials (SBTs) are used to assess the patient’s readiness for liberation from the ventilator [2], and extubation is deemed successful if mechanical assistance is not needed for 48 hours after removal of the endotracheal tube [3]. Extubation is the culmination of the weaning process, and the decision to extubate is usually based on objective parameters demonstrating the patient’s ability to maintain respiratory needs without the aid of a respiratory prosthesis and a mechanical ventilator [4]. Unfortunately, 5%–30% of extubations fail [5–9]. Patients who fail extubation are seven times more likely to die and 31 times more likely to need prolonged ICU stay (≥14 days) than patients with successful extubation [9].

Several parameters have been used to predict the patient’s fitness for weaning from mechanical ventilation and extubation [4]. However, no single parameter can reflect the status of different organs and systems; respiratory parameters alone will not suffice because the factors determining success of weaning are complex and diverse, and vary from patient to patient [3]. Different multifactor scores and indices have been created for this purpose [10–12], but none of them can differentiate prediction of weaning success (success in SBT) from extubation success (removal of the endotracheal tube and absence of mechanical assistance for 48 hours); such differentiation is essential, given the differences in the pathophysiology of weaning failure versus extubation failure [1], and the clinical implications, since patient who failed the weaning, back to the IMV, rest for at least 24 hours, and then, can be submitted to another SBT, while extubation failure decisively impact the patients’ prognosis [9].

The purpose of this study was to 1) identify the respiratory and nonrespiratory parameters associated with post-extubation outcome, 2) use these parameters to develop an Extubation Predictive Score (ExPreS) that could be applied in the ICU to predict extubation outcome in patients receiving IMV, and 3) analyze the performance of the proposed score and validate it in a prospective cohort.

## Methods

This prospective observational study was conducted in a university hospital in southern Brazil. The derivation cohort was collected between December 2017 and March 2019, and the validation cohort between July 2019 and June 2020. The local ethics committee (Comitê de Ética em Pesquisa da UNOESC/HUST) waived the requirement for informed consent and approved the study (n° 3.040.334; CAAE: 1170718.5.0000.5367), which was performed in accordance with established ethical standards and the data were analyzed anonymously.

### Participants

**Fig 1:**
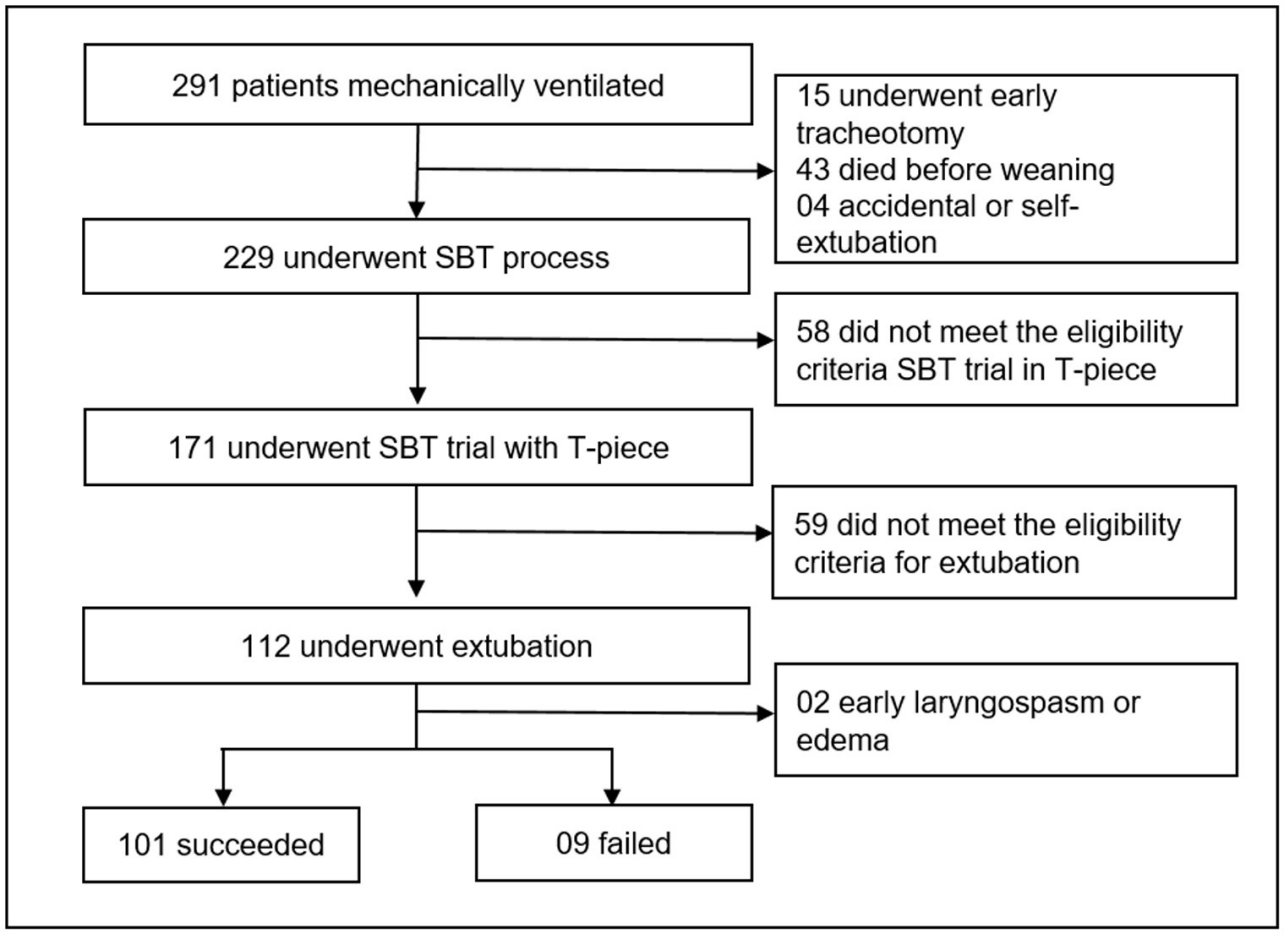
Flowchart of the derivation cohort. *SBT*: Spontaneous Breathing Trial.

Derivation Cohort: All patients aged ≥18 years who were admitted to ICU and submitted to IMV through an endotracheal tube for more than 24 hours were eligible for inclusion. Patients likely to require tracheostomy, having accidental extubation or self-extubation, and not meeting the eligibility criteria for extubation were excluded (Fig 1).

The weaning process followed the established protocol at our ICU (S1 Fig). The first step was the identification of patients ready for weaning; this assessment was based on clinical criteria. The weaning process was started in the pressure-support ventilation (PSV) mode and was followed by a SBT in T-piece for 30 minutes. For patients who passed SBT, the rapid shallow-breathing index (RSBI) was calculated. Those with RSBI <105 breaths/min/L before extubation underwent the cuff-leak test.

Validation Cohort: The patients’ inclusion criteria and weaning protocol followed those used in the derivation cohort. After SBT, the ExPreS was applied and the decision to extubate was made accordingly.

### Data collection

Clinical and demographic data were collected from the medical records. The clinical signs and ventilator parameters during mechanical ventilation (MV) in PSV mode were recorded; MV was administered with inspiratory pressure 7 cm H_2_O over positive end-expiratory pressure (PEEP). Dynamic lung compliance was calculated as tidal volume (mL)/(peak pressure—PEEP), and RSBI was calculated as: total respiratory rate in MV/tidal volume (L). Patients who could tolerate this ventilation were submitted to SBT for 30 minutes and, at the end of this period, the clinical signs and respiratory parameters were recorded again. RSBI was calculated as: respiratory rate in SBT/tidal volume (L), measured by a ventilometer connected to the endotracheal tube.

Muscle strength was graded on a six-point Medical Research Council (MRC) scale [13]. Consciousness level was graded using the estimated Glasgow Coma Score (eGCS) proposed by Meredith et al. (1998) [14].

### Data analysis

The primary outcome of this study was extubation success at 48 hours, i.e., the capacity to maintain spontaneous ventilation for 48 hours after extubation. Extubation failure was defined as the need for reintubation or for institution of noninvasive ventilation during the 48 hours after extubation (the post-extubation respiratory failure was defined when patient presented 2 or more of the following: respiratory rate > 35/min; tidal volume < 5 mL/Kg [based on predicted body weight]; oxygen desaturation despite adequate supplemental oxygen; pH < 7.20 and decreased from onset; hypercapnia with PaCO_2_ > 10 mmHg increased from onset; decreased level of consciousness; abdominal paradox) [15, 16]. Patients who presented early laryngospasm or laryngo/tracheal edema were excluded from the analyses of the derivation cohort to avoid possible bias during the evaluation of the parameters.

Statistical analysis was performed using IBM SPSS statistics, version 25 (IBM Corp., Armonk, NY, USA). Categorical variables were summarized as absolute numbers (n) and relative frequencies (%). The Fisher’s exact test, applied when the contingency was 2 X 2, and chi-square test, applied in all other contingencies, were used to evaluate the association of clinical and demographic characteristics with extubation success or failure. For continuous variables, the Shapiro–Wilk test was used to evaluate the normality of distribution. Normally distributed variables were summarized as means (± standard deviation) and non-normally distributed variables as medians (with interquartile range). The independent samples Student’s *t* test was used to compare differences between groups in normally distributed variables, while the independent samples Mann-Whitney U test to compare differences between groups in non-normally distributed variables.

All parameters with p value lower than 0.4 when in the analyses comparing mean or median values between the extubation success and failure groups were investigated with univariable logistic regression analysis to evaluate their association with extubation outcome; the odds ratios (OR) and 95% confidence intervals (CI) were calculated. Receiver operating characteristics (ROC) analysis was used to assess the predictive value of each parameter. For these parameters, the cutoff values that would provide the optimal balance between sensitivity and specificity were identified using the Youden Index. The parameters with the area under the curve (AUC) higher than 0.5 and neurological comorbidity were selected to be tested in the ExPreS. To determine which parameters would be part of the score, we ordered the parameters from the one with the highest AUC to the one with the lowest AUC, including one by one and calculating the AUC for each resulting combination. The set of parameters that achieved the best AUC values was selected for the proposed ExPreS. In addition, the maximal score assigned to each parameter of the ExPreS was based on the AUC values and the score values for each parameter was directly proportional to the cutoff presented by Youden index. Univariable logistic regression analysis and ROC analysis were used to evaluate ExPreS in comparison with the RSBI (the most used parameter worldwide o predict extubation outcome [4]). The cutoffs of ExPreS and RSBI used in the comparison were stablished based on the Youden Index. Finally, the cutoff ExPreS values indicating low, intermediate, and high probability of extubation success were determined. After SBT, the ExPreS was applied and patients that achieved a score ≥ 59, were extubated; patients with ExPreS lower or equal to 44 were returned to IMV (for at least 24 hours); while patients with ExPreS between 45 and 58, with extubation failure risk factors (obesity [17], cardiopathy [18] and COPD [19]), were extubated and immediately connected to non-invasive mechanical ventilation, while those without these risk factors were extubated following the described protocol (S2 Fig).

## Results

In the derivation cohort, a total of 291 patients were intubated and mechanically ventilated in the ICU during the study period. Of these, 229 underwent the SBT process, and 112 were extubated. Two patients were excluded because they had early laryngospasm or edema. The remaining 110 patients formed the study sample of the derivation cohort; extubation succeeded in 101/110 (91.8%) patients and failed in 9/110 (8.2%) patients (Fig 1).

Age, sex, Acute Physiology and Chronic Health Evaluation Classification System II score (APACHE II score), and diagnoses at ICU admission were comparable between the success and failure groups. Duration of ICU stay was significantly longer in the extubation group (median: 13 days vs. 7 days; p = 0.005; Table 1).

**Table 1:** Patient characteristics—Derivation cohort.

	Total (110)	Success (101)	Failure (09)	p
Age, years**	67 (50–77)	67 (50–67)	69 (48–80)	0.806
Male n (*%*)* §	62 (56)	59 (58)	3 (33)	0.135
APACHE II score, points**	17 (13–21)	17 (13–21)	18 (16–23)	0.358
Admission Diagnosis n (*%*)* §§				
Respiratory Disease	30 (27)	27 (90)	3 (10)	0.666
Post Surgery	23 (21)	21 (91)	2 (9)	
Sepsis or Septic Shock	17 (15)	15 (88)	2 (112)	
Trauma	13 (12)	13 (100)	0 (0)	
Neurologic Disease	12 (11)	10 (83)	2 (17)	
Cardiac Disease	3 (3)	3 (100)	0 (0)	
Others	12 (11)	12 (100)	0 (0)	
ICU length of stay (*days*)**	7 (6–10)	7 (5–9)	13 (8–20)	0.005

*presented as absolute and relative frequency.

**presented as median and interquartile range and applied Mann-Whitney U test.

^§^ Fisher Exact test.

^§§^ Chi-square test.

ICU: Intensive Care Unit.

Significant differences were seen between extubation success and failure groups in some clinical parameters related to the extubation outcome (Table 2). Neurologic comorbidities were more common in the extubation failure group (66.7% vs. 27.7%; p = 0.024). Dynamic lung compliance was lower in the extubation failure group (median: 44.2 mL/cm H_2_O vs. 56.2 mL/cm H_2_O; p = 0.047). Tidal volume in MV was lower in the extubation failure group (median: 6.4 mL/kg vs. 8.1 mL/kg; p = 0.016). RSBI in MV and at the end of SBT was higher in the extubation failure group (median: 57.5 breaths/min/L vs. 38 breaths/min/L; p = 0.003 and 62.8 breaths/min/L vs. 47.5 breaths/min/L; p = 0.006; respectively).

**Table 2:** Comparison of parameters between the extubation success and failure group in the derivation cohort.

	Success (101)	Failure (09)	p
Respiratory Comorbidity *	45 (45)	5 (55)	0.729
Cardiac Comorbidity *	24 (24)	1 (11)	0.681
Neurologic Comorbidity *	28 (27.7)	6 (67)	**0.024**
Two or more comorbidities *	22 (22)	4 (44)	0.211
SOFA score **	5 (4–6)	5 (4–8)	0.356
Days of Mechanical Ventilation**	5 (2–7)	5 (4–10)	0.064
Days of Sedation**	3 (2–10)	3 (1–4)	0.522
Estimated Glasgow Coma Scale**	14.7 (13.3–14.7)	14.3 (8.8–14.7)	0.136
Muscle Strength (MRC) **	48 (48–54)	48 (24–48)	0.081
Fluid Balance—last 24h (ml) **	510 (-365–1625)	576 (-154–1176)	0.831
Fluid Balance—ICU stay (ml) **	3876 (1088–8411)	4435 (281–7530)	0.802
Creatinine (mg/dL) **	0.86 (0.68–1.71)	1.23 (0.7–2.45)	0.386
Urea (mg/dL) **	52 (34.6–81.4)	54.2 (43.9–98)	0.549
Hematocrit (%) ***	29.1 ± 5.7	25.7 ± 7.4	0.100
Dynamic Lung Compliance (ml/cmH2O) **	56.2 (43.7–66.1)	44.2 (43.7–48.8)	**0.047**
Arterial pH**	7.47 (7.39–7.53)	7.49 (7.44–7.53)	0.520
Arterial PO2 (mmHg) **	85.2 (66.6–125.5)	103.1 (85.5–125.5)	0.346
Arterial PCO2 (mmHg) **	31.3 (26.4–34)	30.2 (27.7–33.6)	0.896
HR in MV **	85 (71.5–95.5)	87 (74.5–98.5)	0.739
HR in SBT **	85 (69.2–97)	80 (69–94)	0.628
SatO2 (%) in MV **	97 (96–98)	98 (97–99)	0.398
SatO2 (%) in SBT **	97 (95–98)	98 (96–99)	0.163
RR in MV **	20 (16–23.5)	23 (19–23.5)	0.125
RR in SBT **	21 (17–23.7)	22 (18.5–27.5)	0.310
Tidal Volume (ml/Kg) in MV **	8.1 (7–10.1)	6.4 (5.9–7.6)	**0.016**
Tidal Volume (ml/Kg) in SBT **	7.2 (6–8.4)	6.4 (4.2–7.6)	0.098
RSBI in MV (breaths/min/L) **	38 (29.5–50.9)	57.5 (48–66.3)	**0.003**
RSBI in SBT (breaths/min/L) **	47.5 (36.6–58.2)	62.8 (51.5–87)	**0.006**

*presented as absolute and relative frequency and applied Fisher Exact test.

**presented as median and interquartile range and applied Mann-Whitney U test.

***presented as mean ± standard deviation and applied Student t test.

SOFA: Sequential Organ Failure Assessment. MRC: Medical Research Council. ICU: Intensive Care Unit. HR: Heart Rate. MV: Mechanical Ventilation. SBT: Spontaneous Breathing Trial. RR: Respiratory Rate.

Univariable logistic regression analysis was performed for those clinical parameters with potential to contribute to the proposed multiparameter score. The parameters associated with increased risk of extubation failure were the following: presence of a neurologic comorbidity (OR = 5.21, p = 0.026), long duration of MV (OR = 1.28, p = 0.045), and high RSBI in MV (OR = 1.04, p = 0.021) and in SBT (OR = 1.06, p = 0.005). The parameters associated with decreased risk of extubation failure included the following: high eGCS (OR = 0.57, p = 0.003) and high tidal volume in MV (OR = 0.60, p = 0.022). Four other parameters that did not show statistically significant association with extubation outcome were also tested in the ROC analyses; these included muscle strength (OR = 0.52, p = 0.054), hematocrit (OR = 0.90, p = 0.104), dynamic lung compliance (OR = 0.95, p = 0.063), and serum creatinine (OR = 1.22, p = 0.262) (Table 3).

**Table 3:** Potential predictors of extubation failure based in an univariable logistic regression analysis (derivation cohort).

	OR	95% CI	p
Neurologic Comorbidity	5.21	1.22–22.29	**0.026**
Two or more comorbidities	2.87	0.71–11.62	0.139
SOFA score	1.11	0.87–1.41	0.388
Days of Mechanical Ventilation	1.28	1.01–1.63	**0.045**
Estimated Glasgow Coma Scale	0.57	0.39–0.82	**0.003**
Muscle Strength (MRC)	0.52	0.27–1.01	0.054
Creatinine (mg/dL)	1.22	0.86–1.71	0.262
Hematocrit (%)	0.90	0.79–1.02	0.104
Dynamic Lung Compliance (ml/cmH2O)	0.95	0.90–1.00	0.063
Arterial PO2 (mmHg)	1.00	0.99–1.02	0.732
SatO2 (%) in MV	1.06	0.74–1.53	0.733
SatO2 (%) in SBT	1.11	0.82–1.50	0.502
RR in MV	1.02	0.96–1.10	0.464
RR in SBT	1.01	0.95–1.08	0.664
Tidal Volume (ml/Kg) in MV	0.60	0.39–0.93	**0.022**
Tidal Volume (ml/Kg) in SBT	0.63	0.39–1.02	0.059
RSBI in MV (breaths/min/L)	1.04	1.01–1.08	**0.021**
RSBI in SBT (breaths/min/L)	1.06	1.02–1.10	**0.005**

OR: odds ratios. CI: 95% confidence intervals. SOFA: Sequential Organ Failure Assessment. MRC: Medical Research Council. MV: Mechanical Ventilation. SBT: Spontaneous Breathing Trial. RR: Respiratory Rate. RSBI: Rapid shallow-breathing index.

**Fig 2:**
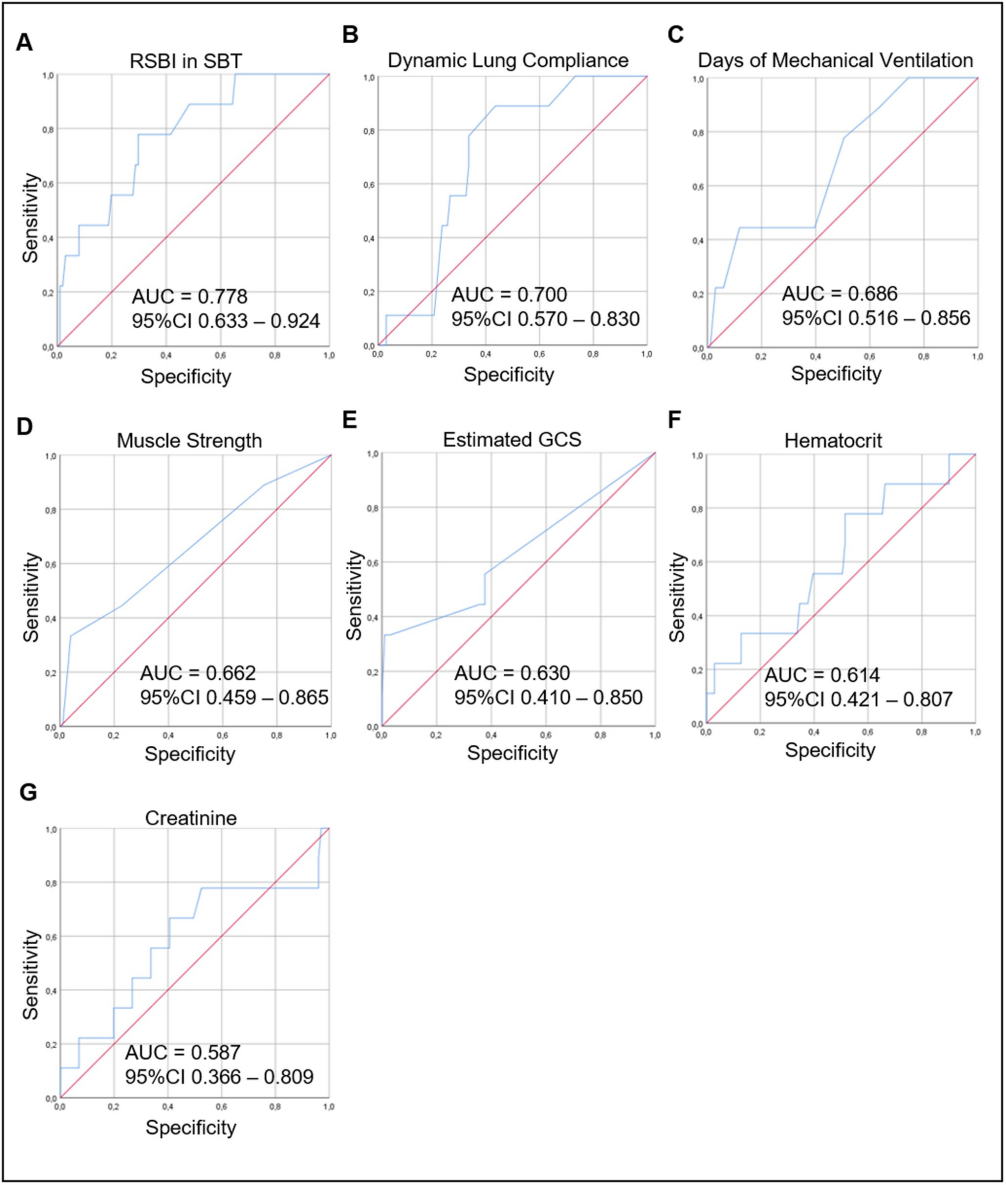
ROC curves for RSBI in SBT (A), Dynamic Lung Compliance (B), Days of Mechanical Ventilation (C), Muscle Strength (D), Estimated GCS (E), Hematocrit (F), and Creatinine (G) used to distinguish the extubation success group from the failure group. In the ROC curve analysis, the AUC was highest for RSBI in SBT (0.778), followed by the Dynamic Lung Compliance (0.700), Days of Mechanical Ventilation (0.686), Muscle Strength (0.662), Estimated GCS (0.630), Hematocrit (0.614), and Creatinine (0.587). ROC curve: receiver operating characteristic curve; RSBI in SBT: Rapid Shallow Breathing Index in Spontaneous Breathing Trial; Estimated GCS: Glasgow Coma Scale; AUC: area under the curve, CI: confidence interval.

Next, we assessed the ability of each parameter to distinguish between the extubation success and failure groups. As Fig 2 shows, the highest AUC was found for RSBI in SBT (0.778), followed by dynamic lung compliance (0.700), duration of MV (0.686), muscle strength (0.662), eGCS (0.630), hematocrit (0.614), and serum creatinine (0.587). Table 4 presents the cutoff value for the highest Youden index for each of these clinical parameters.

**Table 4:** Sensitivity and specificity at the cutoff of the highest Youden Index—Derivation cohort.

	Cutoff	Sensitivity	Specificity
RSBI in SBT (breaths/min/L)	55	0.778	0.703
Dynamic Lung Compliance (ml/cmH2O)	51	0.889	0.564
Days of Mechanical Ventilation	4	0.778	0.495
Muscle Strength (MRC)	48	0.444	0.772
Estimated Glasgow Coma Scale	14	0.556	0.624
Creatinine (mg/dL)	1.0	0.667	0.594
Hematocrit (%)	29	0.778	0.485

RSBI: Rapid shallow-breathing index. SBT: Spontaneous Breathing Trial. MRC: Medical Research Council.

**Fig 3:**
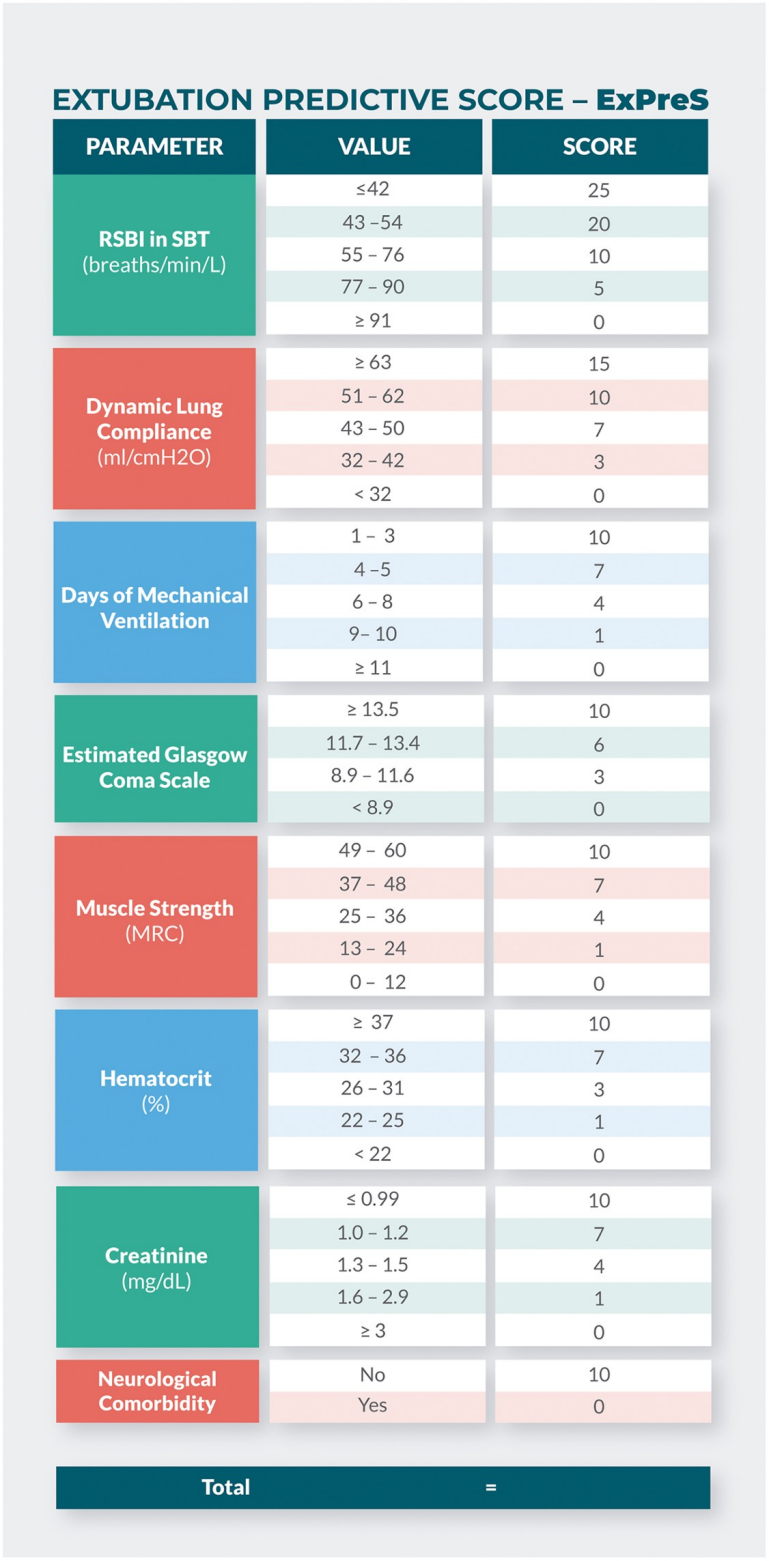
Extubation Predictive Score—ExPreS.

**Fig 4:**
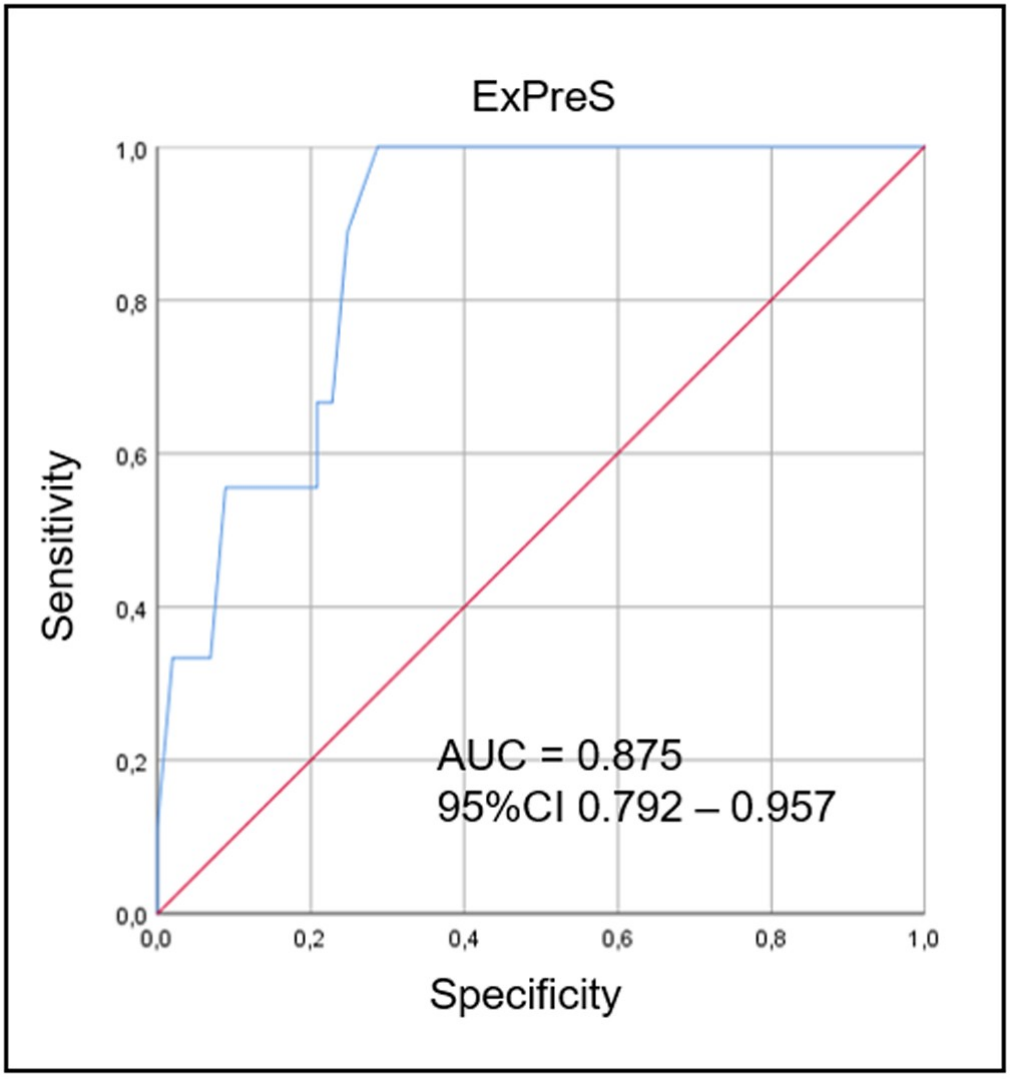
ROC curves for ExPreS from derivation cohort, used to distinguish the extubation success group from the failure group. In the ROC curve analysis, the AUC was 0.875. ROC curve: receiver operating characteristic curve; ExPreS: Weaning and Extubation Predictive Index; AUC: area under the curve, CI: confidence interval.

The parameters with the best AUC values, over 0.5 (RSBI in SBT, dynamic lung compliance, duration of MV, eGCS, muscle strength, hematocrit and serum creatinine), beyond the neurological comorbidity, were tested one by one, and the set that achieved the higher AUC value were incorporated in the ExPreS (Fig 3). The total score of ExPreS can vary from 0 to 100. RSBI in MV is the parameter with the highest maximum score (25 points), followed by dynamic lung compliance (15 points); for all other parameters, the maximum score is 10 points. Univariable logistic regression (S1 Table) and ROC analyses showed that the ExPreS had better predictive capability than any of the individual parameters tested; the AUC for ExPreS was 0.875 (Fig 4).

**Fig 5:**
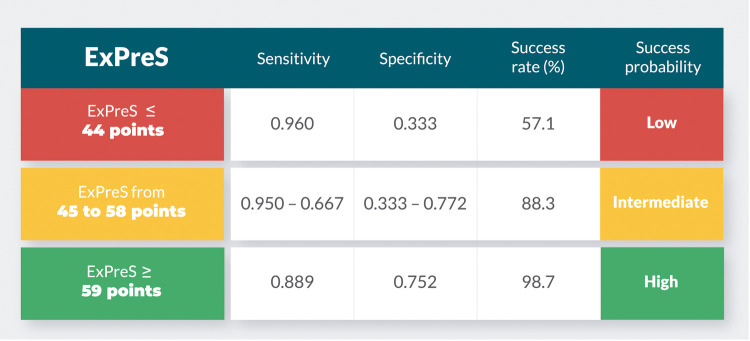
Extubation success probability of ExPreS. The sensitivity, specificity and success rate (%) of ExPreS ≤ 44 points, ExPreS from 45 to 58 points, and ExPreS ≥ 59 points.

Using Youden Index from the ROC analysis, we determined the cutoff values of RSBI in SBT and ExPreS for prediction of extubation success. The OR for extubation success was 4.43 (p = 0.168) for patients with RSBI in SBT <43 breaths/min/L. The OR for extubation success was 23.07 (p = 0.004) for patients with ExPreS ≥59 points and 0.82 (p = 0.004) for patients with ExPreS ≤44 points (S2 Table). Fig 5 presents the extubation success probability according to the ExPreS. Patients with ExPreS ≤ 44 points had low probability of extubation success (success rate = 57.1%; Positive Predictive Value [PPV] = 97% and Negative Predictive Value [NPV] = 23%); patients with ExPreS score 45–58 points had intermediate probability of extubation success (success rate = 83.3%); and patients with ExPreS ≥59 points had high probability of extubation success (success rate = 98.7%; PPV = 97% and NPV = 11%).

**Fig 6:**
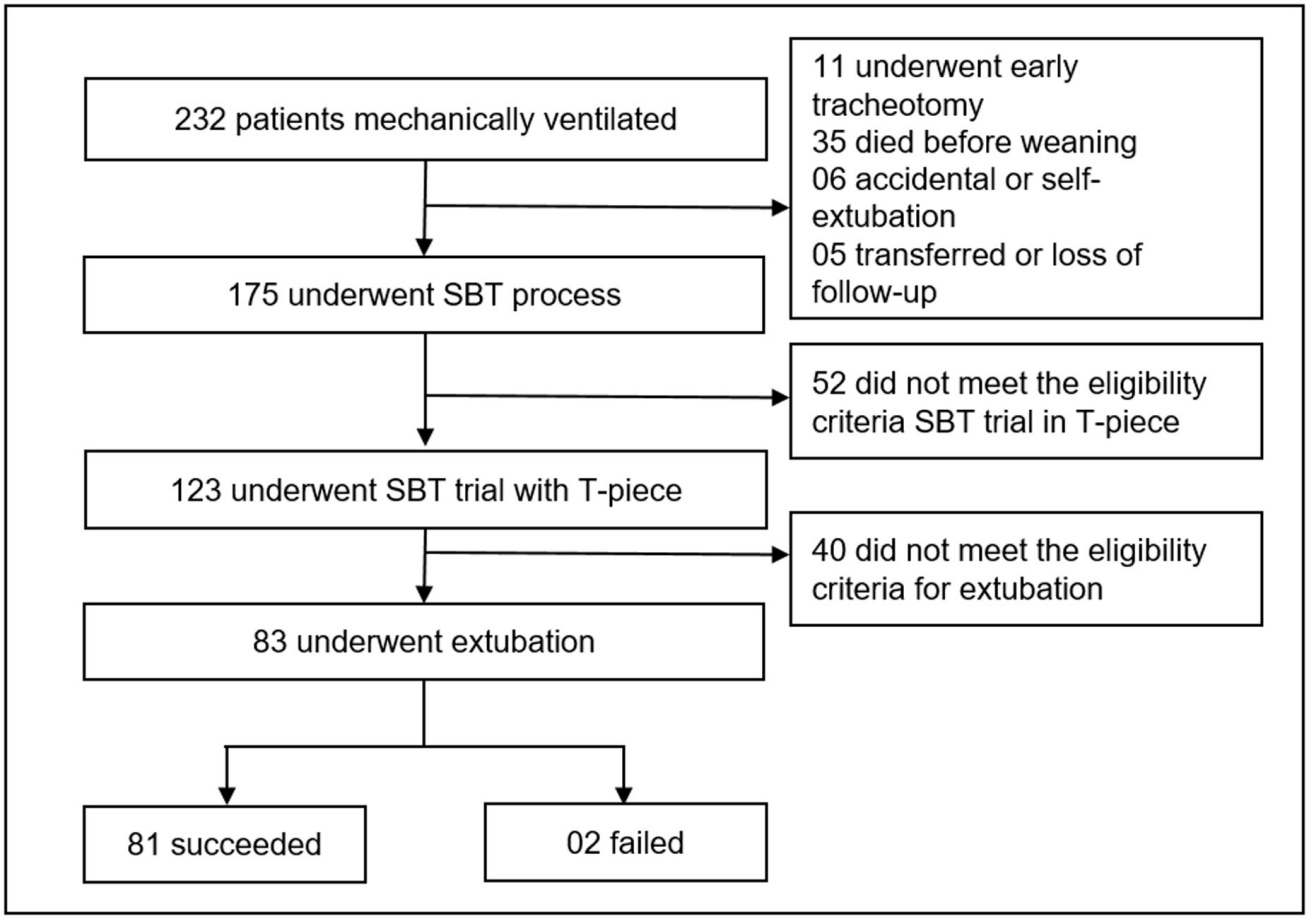
Flowchart of the validation cohort. *SBT*: Spontaneous Breathing Trial.

**Fig 7:**
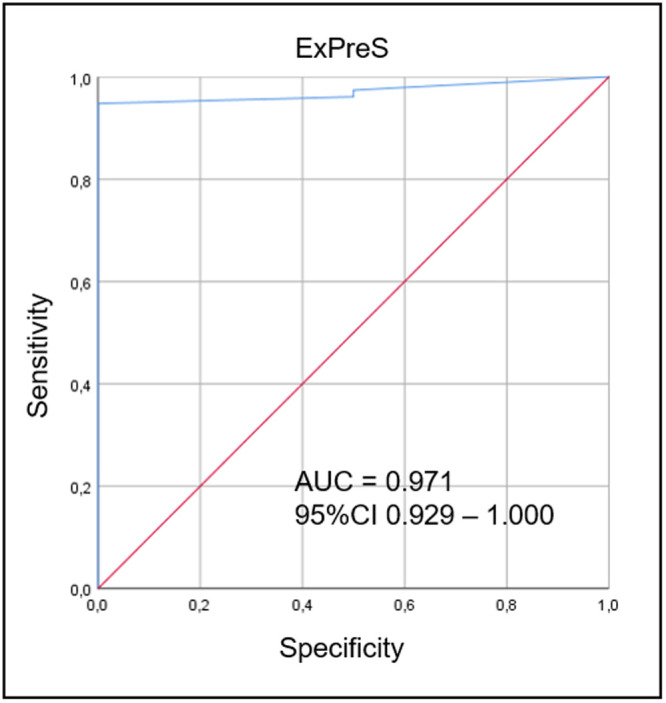
ROC curves for ExPreS from validation cohort, used to distinguish the extubation success group from the failure group. In the ROC curve analysis, the AUC was 0.971. ROC curve: receiver operating characteristic curve; ExPreS: Weaning and Extubation Predictive Index; AUC: area under the curve, CI: confidence interval.

In the validation cohort, 232 patients were intubated and mechanically ventilated in the ICU. Of these, 175 underwent the SBT process, and 83 were extubated forming the validation cohort; extubation succeeded in 81/83 (97.6%) patients and failed in 2/83 (2.4%) patients (Fig 6). No patient with ExPreS ≥ 59 failed extubation. Patients who failed had ExPreS of 51 and 54 points, with the latter patient being reintubated due to laryngospasm, which indicates a failure in the cuff-leak test, not in the ExPreS. Comparing validation and derivation cohort, no significant difference in characteristics as age, sex, admission diagnosis and in the duration of the mechanical ventilation was observed. In the derivation cohort the extubation success rate was 91.8% in patients with a median APACHE II score of 17 points and median IMV duration of 5 days, while in the validation cohort the extubation success rate was 97.6%, the median APACHE II score was 23.5 points (characterizing more severe patients at admission compared to derivation cohort) and with the same median IMV duration (Table 5). Despite that, the use of ExPreS decreased the extubation failure rate from 8.2% to 2.4%, (p = 0.1191) and the AUC for ExPreS in the validation cohort was 0.971 (Fig 7).

**Table 5:** Patient characteristics by cohort.

	Derivation Cohort (110)	Validation Cohort (83)	p
Age, years**	67 (50–77)	62 (48–72)	0.08
Male n (*%*)*§	62 (56)	43 (52)	0.561
APACHE II score, points**	17 (13–21)	23.5 (17–29)	0.000
Admission Diagnosis n (*%*)*§§			
Respiratory Disease	30 (27)	15 (18)	0.277
Post Surgery	23 (21)	24 (29)	
Sepsis or Septic Shock	17 (15)	9 (11)	
Trauma	13 (12)	9 (11)	
Neurologic Disease	12 (11)	12 (14)	
Cardiac Disease	3 (3)	7 (8)	
Others	12 (11)	7 (8)	
Days of Mechanical Ventilation, days**	5 (2–7)	5 (2.5–8.5)	0.182

*presented as absolute and relative frequency.

**presented as median and interquartile range and applied Mann-Whitney U test.

^§^ Fisher Exact test.

^§§^ Chi-square test.

## Discussion

In this study, we observed that the ExPreS, a multiparameter score that we developed by incorporating different respiratory and nonrespiratory parameters associated with extubation outcome, is a reliable predictor of extubation outcome in patients receiving IMV in the ICU. Besides the good statistical power in ExPreS to predict extubation success, the use of ExPreS in the validation cohort decreased the extubation failure rate from 8.2% (derivation cohort) to 2.4%, even though the validation cohort was composed of more severe patients at admission. Of note, all patients with scores in ExPreS higher or equal to 59 presented successful extubations, and of the two patients who failed, one was due to laryngospasm, probably due to the false negative result in the cuff-leak test.

Extubation failure may be associated with worse outcomes [9]. The reintubation and reinstitution of IMV may be associated with worse outcomes not only because of possible complications of this process, but mostly, because patients who fail extubation probably have a worse clinical condition. This highlights the need for an instrument that can reliably identify patients likely to fail extubation.

The aim of this study was to identify the parameters associated with post-extubation outcome, develop a score that could predict extubation outcome in patients receiving IMV, and analyze the performance of the proposed score in a prospective validation cohort.

In the ExPreS we included eight parameters. RSBI and dynamic lung compliance are two important measures of respiratory mechanics [4]. The value of RSBI for predicting weaning and extubation success has been examined by many researchers [4]. RSBI <105 breaths/min/L was initially considered to be predictive of extubation success [1]; however, subsequent studies showed that values around 50 breaths/min/L are associated with extubation success, while values around 80 breaths/min/L are associated with failure [20, 21]. In the present study, the median RSBI in SBT was 47.5 breaths/min/L in the extubation success group vs. 62.8 breaths/min/L in the failure group; the AUC was 0.778 (the highest among the eight parameters).

Dynamic lung compliance reflects the lung’s capacity to expand. Consistent with previous studies [10, 22], we found higher dynamic lung compliance in patients who succeed extubation than in patients who fail extubation.

IMV duration was another parameter that differed between the extubation success and failure groups. This was probably because the combination of complete diaphragmatic inactivity and MV results in atrophy of diaphragm myofibers. Such atrophy has been reported even after only 18 hours of IMV [18]. The longer the duration of IMV, the higher the risk of ventilator-associated complications, morbidity, and mortality, the higher the hospitalization costs [1], and the lower the chance of success in weaning and extubation [10, 20, 23, 24].

Level of consciousness and muscle strength are parameters that reflect the general condition of the patient. Consciousness level—evaluated by GCS [10] and modified GCS [19]—has been found to be associated with weaning [23] and extubation [10] outcomes. However, since it is not possible to assess verbal response in intubated patients, in this research we used the eGCS, which estimates the verbal score based on the GCS eye opening and motor scores [14]. We found that the eGCS could be useful for predicting extubation outcome. Similarly, loss of peripheral muscle strength correlates with loss of the respiratory muscle strength [25]; a previous study has shown that MRC score >41 can predict weaning success [26].

Serum creatinine level and hematocrit were two other parameters incorporated in the ExPreS. Both are routinely evaluated in the ICU setting. Blood urea nitrogen [23, 27] and need for hemodialysis [28] are other measures of renal function that have also been reported to be predictive of weaning and extubation failure. Among the consequences of kidney failure is the inability to eliminate fluids; this can lead to pulmonary congestion. While inability to maintain fluid balance has been previously found to be related to extubation failure [20, 29], in the present cohort it was not associated with extubation outcome. In previous studies, hemoglobin was reported to be related to weaning [30] and extubation [29, 31] outcome. We measured hematocrit in the present study and found that it was related extubation outcome. Anemia can exacerbate the insufficient global oxygen delivery observed in patients who fail weaning [32].

In the present study, among the comorbidities only neurological comorbidity was related to extubation failure; this was probably via impairment of respiratory control and inability to protect the airways [33], which are common in patients on IMV. In previous studies, however, the presence and severity of comorbidities (in general) has been found to be related to extubation outcome [34].

Although previous studies have found age [10, 21, 24, 35], APACHE II score [34, 36], and blood gas data [21, 29] to be associated with extubation outcome, we found no significant association in the present study.

Several multiparameter scores for predicting fitness for weaning and extubation have been proposed earlier, for example, the scores created by Mongaroth [37] in the 70s, and by Gluck and Corgian [38] and Yang and Tobin [39] in the 90s. More recent examples are the CORE index [40], the Integrative Weaning Index (IWI) [11], the Burns Wean Assessment Program (BWAP) [12], and the modified-BWAP [10]. However, these scores have not proved to be very efficient: even with their application, the extubation failure rate remains in the range of 11.5%–21.5% [10–12, 40]. Our hypothesis to explain the better performance of ExPreS in relation to previous scores is the capacity to evaluate the status of different systems, not only the respiratory.

Extubation success rates vary widely in previous studies (5%–30%) [5–9], reflecting differences in weaning and extubation protocols, severity of illness, and ICU specialization. The median APACHE II score and duration of MV in our study are very similar to the values presented by Fernandez el al. (2017) in a study that involved 17 Spanish medical and surgical intensive care units (median APACHE II score: 18.3 and 17.8 points, respectively, and median duration of IMV: 5 days) [2].

In the last few years, ultrasound of the diaphragm and lung has demonstrated good potential to predict weaning outcome; however, the limitations are the need for equipment (which may not be available in all ICUs) and for staff training, and also the accuracy, which may vary with the patient subpopulation [41].

This study presents as limitation the low statistical power in extubation failure prediction due the small sample of patients with extubation failure in our analysis, resulting in the low NPV for both, ExPreS ≤ 44 and ≥ 59 points. This low NPV limits the ExPreS in clinical practice, mainly in determining whether patients with ExPreS < 59 will actually fail extubation. Although our results, based in two cohorts, suggest that clinicians can apply the ExPreS in their clinical practice, we encourage them to conduct Randomized Clinical Trials comparing the application of ExPreS with the standard of care during the extubation process, testing the real accuracy of the ExPreS to predict the extubation outcome.

## Conclusion

The ExPreS, a multiparameter score that we developed by incorporating different respiratory and nonrespiratory parameters associated with extubation outcome, is a reliable predictor of extubation outcome in patients receiving IMV in the ICU. In the validation cohort, the use of ExPreS decreased the extubation failure rate from 8.2% to 2.4%, even in a cohort of more severe patients. It is a simple method and is easily applied at the bedside.

## Supporting information

S1 Fig Flowchart of the weaning and extubation protocol for derivation cohort. *PSV*: Pressure Support Ventilation; *SBT*: Spontaneous Breathing Trial; *RR*: Respiratory Rate; *HR*: Heart Rate; *SBP*: Systolic Blood Pressure; *MV*: Mechanical Ventilation: *RSBI*: Rapid Shallow-Breathing Index. *TVi*: Inspiratory Tidal Volume.
(TIF)

S2 Fig Flowchart of the weaning and extubation protocol for validation cohort. *PSV*: Pressure Support Ventilation; *SBT*: Spontaneous Breathing Trial; *RR*: Respiratory Rate; *HR*: Heart Rate; *SBP*: Systolic Blood Pressure; *MV*: Mechanical Ventilation: *RSBI*: Rapid Shallow-Breathing Index. *TVi*: Inspiratory Tidal Volume. *COPD*: Chronic Obstructive Pulmonary Disease.
(TIF)

S1 Table Odds ratios and confidence intervals for predictors of extubation success based in an univariable logistic regression analysis—Derivation cohort. OR: odds ratios. CI: 95% confidence intervals. RSBI: Rapid shallow-breathing index. SBT: Spontaneous Breathing Trial. ExPreS: Extubation Predictive Score.
(DOC)

S2 Table Odds ratios and confidence intervals for predictors of extubation success based in an univariable logistic regression analysis for cutoff values—Derivation cohort. OR: odds ratios. CI: 95% confidence intervals. RSBI: Rapid shallow-breathing index. SBT: Spontaneous Breathing Trial. ExPreS: Extubation Predictive Score.
(DOCX)

S1 Checklist STROBE statement. (DOC)

S1 Data (XLSX)
