# Supplementary figures and images for: Prediction of extubation outcome in mechanically ventilated patients: Development and validation of the Extubation Predictive Score (ExPreS)
Source: PLoS One. 2021 Mar 18;16(3):e0248868. doi: 10.1371/journal.pone.0248868 (PMC7971695; doi:10.1371/journal.pone.0248868)

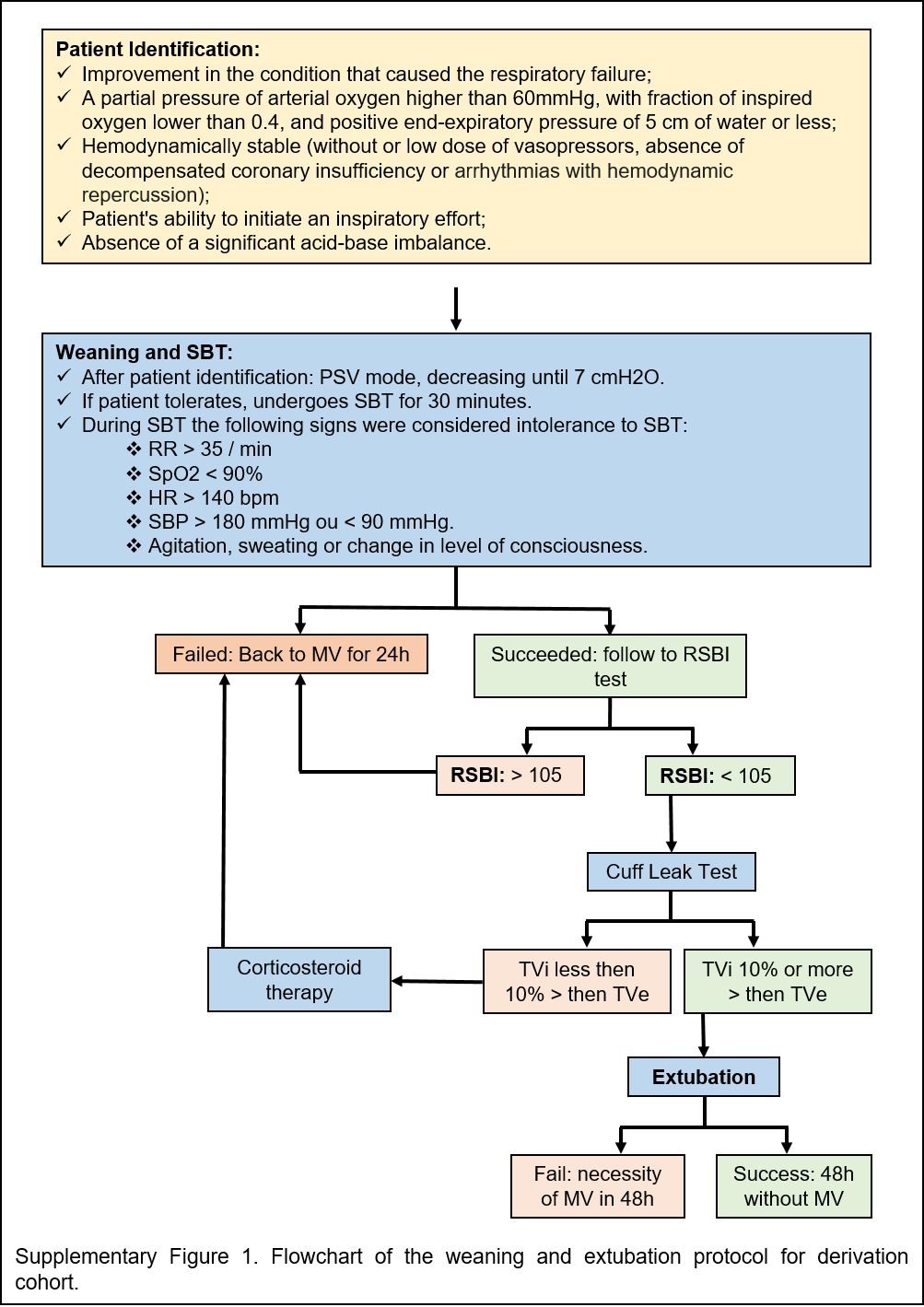

Supplement: S1 Fig — PSV: Pressure Support Ventilation; SBT: Spontaneous Breathing Trial; RR: Respiratory Rate; HR: Heart Rate; SBP: Systolic Blood Pressure; MV: Mechanical Ventilation: RSBI: Rapid Shallow-Breathing Index. TVi: Inspiratory Tidal Volume. (TIF) [file pone.0248868.s001.tif]

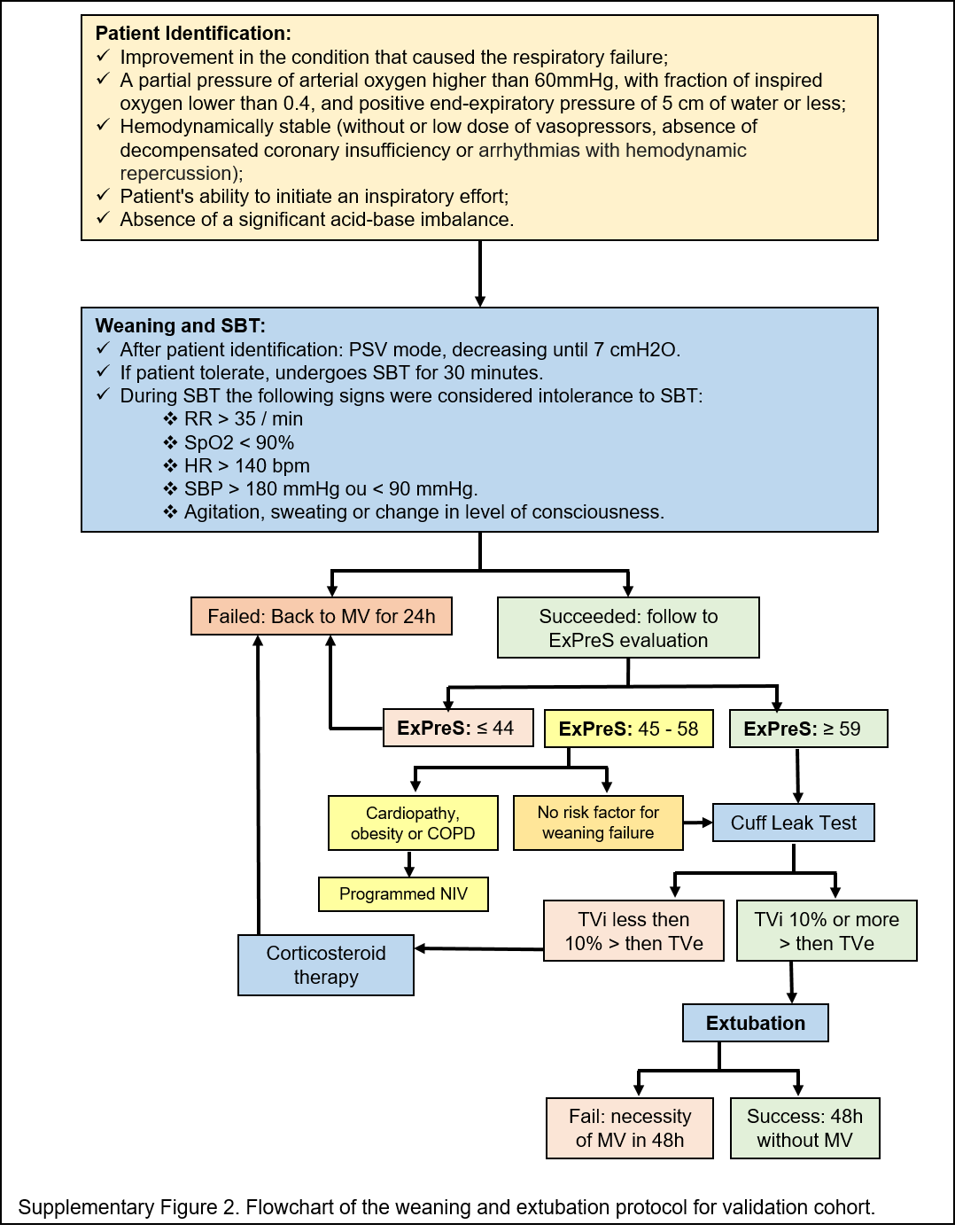

Supplement: S2 Fig — PSV: Pressure Support Ventilation; SBT: Spontaneous Breathing Trial; RR: Respiratory Rate; HR: Heart Rate; SBP: Systolic Blood Pressure; MV: Mechanical Ventilation: RSBI: Rapid Shallow-Breathing Index. TVi: Inspiratory Tidal Volume. COPD: Chronic Obstructive Pulmonary Disease. (TIF) [file pone.0248868.s002.tif]
