# Supplementary material for: Prediction of extubation outcome in mechanically ventilated patients: Development and validation of the Extubation Predictive Score (ExPreS)
Source: PLoS One. 2021 Mar 18;16(3):e0248868. doi: 10.1371/journal.pone.0248868 (PMC7971695; doi:10.1371/journal.pone.0248868)
Supplement: S1 Table — OR: odds ratios. CI: 95% confidence intervals. RSBI: Rapid shallow-breathing index. SBT: Spontaneous Breathing Trial. ExPreS: Extubation Predictive Score. (DOC) [file pone.0248868.s003.doc]

| **S1 Table.** Odds ratios and confidence intervals for predictors of extubation success based in an univariable logistic regression analysis – derivation cohort. | | | |
| --- | --- | --- | --- |
|  | OR | 95% CI | p |
| RSBI in SBT (breaths/min/L) | 1.06 | 1.02 – 1.10 | **0.005** |
| ExPreS | 0.90 | 0.85 – 0.96 | **0.001** |
