# Supplementary material for: Prediction of extubation outcome in mechanically ventilated patients: Development and validation of the Extubation Predictive Score (ExPreS)
Source: PLoS One. 2021 Mar 18;16(3):e0248868. doi: 10.1371/journal.pone.0248868 (PMC7971695; doi:10.1371/journal.pone.0248868)
Supplement: S2 Table — OR: odds ratios. CI: 95% confidence intervals. RSBI: Rapid shallow-breathing index. SBT: Spontaneous Breathing Trial. ExPreS: Extubation Predictive Score. (DOCX) [file pone.0248868.s004.docx]

| **S2 Table.** Odds ratios and confidence intervals for predictors of extubation success based in a univariable logistic regression analysis for cutoff values – derivation cohort. | | | |
| --- | --- | --- | --- |
|  | OR | 95% CI | p |
| RSBI in SBT - cutoff of 43 breaths/min/L | 4.43 | 0.53 – 36.85 | 0.168 |
| ExPreS - cutoff of 44 points | 0.82 | 0.015 – 0.456 | **0.004** |
| ExPreS - cutoff of 59 points | 23.07 | 2.75 – 193.45 | **0.004** |
